# The Use of Natural Language Processing Elements for Computer-Aided Diagnostics and Monitoring of Body Image Perception in Enterally Fed Patients with Head and Neck or Upper Gastrointestinal Tract Cancers

**DOI:** 10.3390/cancers16071353

**Published:** 2024-03-29

**Authors:** Agnieszka Różańska, Elwira Gliwska, Klaudia Barańska, Stella Maćkowska, Adrianna Sobol, Dominik Spinczyk

**Affiliations:** 1 Faculty of Biomedical Engineering, Silesian University of Technology, 41-800 Zabrze, Poland; 2 Department of Food Market and Consumer Research, Institute of Human Nutrition Sciences, Warsaw University of Life Sciences (WULS-SGGW), 02-776 Warsaw, Poland; 3 Maria Sklodowska-Curie National Research Institute of Oncology, Polish National Cancer Registry, 02-034 Warsaw, Poland; 4 Department of Oncological Propaedeutics, Medical University of Warsaw, 01-445 Warsaw, Poland

**Keywords:** body image perception, computer-aided diagnostics, computer-aided diagnostics and monitoring, natural language processing, head and neck cancer, upper gastrointestinal tract cancer

## Abstract

**Simple Summary:**

Cancer stands as a leading cause of death globally and remains a significant threat to public health in developed nations. With an anticipated rise in cancer cases, the number of survivors is also expected to increase, many of whom grapple with distorted body image perception. Addressing the common poor quality of life among cancer patients and survivors necessitates monitoring emotions and body perception to mitigate psychological burdens. Given the strain on healthcare resources, exploring automated screening methods for cancer patients is crucial. This article proposes leveraging Natural Language Processing for monitoring body image perception in enterally fed patients with head and neck or upper gastrointestinal tract cancers, offering potential advancements in care delivery and patient support within oncology settings.

**Abstract:**

Background: Psycho-oncology care has emerged as a significant concern in contemporary oncology practice, given its profound impact on patient psychological well-being. Patients undergoing treatment for head–neck or upper gastrointestinal tract cancers often experience complex emotional and psychological challenges, necessitating specialized support and intervention. Traditional approaches to psycho-oncological care may be limited in their ability to comprehensively assess and address patients’ needs. Therefore, exploring innovative methodologies, such as leveraging natural language processing (NLP) elements, is crucial to enhancing the effectiveness of psycho-oncological interventions. Methods: In this study, we developed a method utilizing natural language processing (NLP) elements to augment psycho-oncological care for patients with head–neck or upper gastrointestinal tract cancers. The method aimed to facilitate vocabulary, sentiment, and intensity analysis of five basic emotions (happiness, sadness, anger, disgust, and fear), as well as to explore potential areas of difficulty such as body image, pain, and self-esteem. We conducted research involving 50 patients across three treatment stages. Results: Our method facilitated the identification of characteristic features at each treatment stage, aiding in the tailoring of appropriate therapies to individual patient needs. The results offer insights valuable to psychologists and psychiatrists for expedited diagnosis and intervention, potentially influencing therapy outcomes. Additionally, the data may inform treatment decisions by addressing patient-specific concerns. Furthermore, our method holds promise for optimizing the allocation of psychological care resources, particularly at the initial stages of patient contact. Limitations: The main problem in the research was the fairly wide age range of participants, which explains the potential diversity of vocabulary. Conclusion: In conclusion, our study demonstrates the potential utility of integrating natural language processing (NLP) elements into psycho-oncological care for patients with head–neck or upper gastrointestinal tract cancers. The developed method offers a novel approach to comprehensively assessing patients’ emotional states and areas of difficulty, thereby facilitating tailored interventions and treatment planning. These findings underscore the importance of continued research and innovation in psycho-oncology to enhance patient care and outcomes.

## 1. Introduction

Enteral nutrition is one of the most critical nutritional treatment methods in oncology, as ensuring an adequate supply of nutrients is essential for maintaining optimal nutritional status and immune system functions, improving treatment tolerance, and increasing survival rates [1]. Since malnutrition affects the vast majority of cancer patients, we can expect that many patients will require support through enteral nutrition or other forms of treatment [2].

Enteral nutrition involves administering a formula directly to the gastrointestinal tract below the esophagus, either to the stomach or small intestine. Enteral feeding is recommended if a patient can no longer meet their nutritional needs with their usual diet and is diagnosed with malnutrition or is at a high risk of developing malnutrition. The main indications for enteral feeding in oncology are swallowing difficulties, after-treatment complications, and odynophagia. Nutrition is administered via a feeding tube inserted into the stomach through the nasal cavity or externally through the abdominal wall. External access allows for the provision of a nutritional formula directly to the stomach via percutaneous endoscopic gastrostomy (PEG) or to the small intestine using PEG-Percutaneous Endoscopic Jejunostomy (PEG-PEJ) [3].

Due to swallowing disorders caused by tumors in the headstage and neck area or after-treatment consequences, patients are often unable to eat orally, or their feeding is insufficient, which is an additional burden. The need for enteral nutrition (PEG and PEG-PEJ) might also be associated with complications such as aspiration, tube misplacement or dislodgment, refeeding syndrome, medication-related complications, fluid imbalance, insertion-site infection, and agitation [4,5]. Nonetheless, enteral nutrition can significantly support patient nutritional status and improve anticancer treatment outcomes. However, providing all necessary nutrients via feeding tubes is an additional burden on patients and requires specific knowledge and active engagement in providing formulas. The presence of equipment that enables the administration of the mixture directly into the gastrointestinal tract, which is permanently installed in the patient’s body, may distort body image and possibly influence the quality of life. Body image might also be disturbed due to cancer-related complications such as hair loss, dry skin, post-surgery wounds, or significant weight loss [6,7]. Therefore, cancer patients are at high risk of body image disturbances and related mood deterioration. Moreover, food has more than just nutritional functions in human life, and its social, cultural, and psychological aspects should be considered [8]. As such, losing the ability to eat orally may significantly impact cancer patients’ well-being or social lives.

Computer-aided psychology and psychiatry support are current challenges in natural language processing (NLP). One of its potential applications is the assessment of body image in oncological patients. Body image disorders can reduce the quality of life and be one of the first symptoms of low mood or depression. A quick screening assessment of a patient’s emotions over their perception of their own body may allow for early intervention and referral to an appropriate specialist. Due to the general burden of oncological diseases on the healthcare system and society as a whole, it seems reasonable to seek quick and effective methods of assessing patient emotions and detecting mood disorders at an early stage.

The proposed structured NLP methods are a meaningful contribution to supporting the sharing of psycho-oncological patient care among first-contact medical staff. It is minimally invasive and provides a rapid diagnosis of the patient’s condition by determining the nature of their vocabulary, attitude toward their body, the intensity of accompanying basic emotions, and potential areas of difficulty.

This article is a continuation of the authors’ previous article published in this journal [9]. A group of 50 patients was divided into three stages of treatment, which correlate with the duration of the illness: stage 1, stage 2, and palliative care patients. The novelty of this publication lies in the sentiment analysis conducted, taking into account each of the mentioned patient groups. In the context of areas of difficulty, similarly to previous studies, particular attention was devoted to examining body image perception and self-assessment, recognizing these areas of difficulty as crucial from the perspective of psychological interventions for patients affected by this issue. The decision was made to omit the analysis of environmental acceptance in favor of a new area of difficulty, which is experiencing pain. An analysis of the results regarding areas of difficulty was performed based on each of the aforementioned stages of the illness. Additionally, an analysis of the average values of both emotions and areas of difficulty was conducted, considering the division into groups of patients experiencing pain and those who did not. In relation to the previous article, the transformations that occur between moving to subsequent stages of the disease were analyzed. A theoretical description of the subsequent stages of the disease was published in the above-mentioned article. Moreover, in the current paper, statistical tests were performed between the method results and the expert flags. The second analysis focused on the relationship between body image and emotions and on differentiating the stages of the disease based on emotions.

## 2. Materials and Methods

Section 2.1 and Section 2.2 describe the research materials and methods used.

### 2.1. Materials

The study included 50 patients (26 females and 24 males) with a mean age of 57 years (age range 25–75 ± 11.2). All participants were diagnosed with cancer of the upper gastrointestinal tract or head and neck area and required enteral nutrition. Malignant neoplasms were identified according to the International Statistical Classification 10 [ICD 10] classification and affected other and unspecified parts of the tongue (*n* = 1), the palate (*n* = 4), the parotid gland (*n* = 4), other and unspecified major salivary glands (*n* = 1), the tonsils (*n* = 7), the oropharynx (*n* = 9), the esophagus (*n* = 5), the accessory sinuses (*n* = 5), the larynx (*n* = 7), bone and articular cartilage of other and unspecified sites (*n* = 1), the cerebrum, except lobes and ventricles (*n* = 4), and the thyroid gland (*n* = 2). Patients were under oncological care and treatment between April and December 2021. To be included in the study, a person had to have good verbal and logical communication and give informed consent to participate.

Body image assessment used open questions, to which respondents were instructed to reply using at least two written or spoken sentences. The first question was, *“how do you perceive your body?*”. Further explanations were provided if needed, such as “*describe in your own words how you perceive your body or describe what your body looks like*”. The Bioethics Committee of the Medical Center for Postgraduate Education in Warsaw, Poland, approved the study by resolution on 14 July 2021, ordinance 116/2018. All methods were performed in accordance with the relevant guidelines and regulations.

### 2.2. Methods

#### 2.2.1. Text Analysis

An analysis of patient notes on the characteristics of the language they use and the quality and type of specific word classes can constitute a linguistic portrait of an individual. From a psychological point of view, this may help tailor therapy to a patient’s current condition and needs. After analyzing such patient notes and consulting with an expert in psycho-oncology, we aimed to ascertain the quality of their language, attitude toward their body, and frequency of using specific word classes.

The analysis involved calculating the frequency of using a particular part of speech, such as verbs, verb tenses (present or past references), and adjectives (positive and negative). Next, the study measured the frequency of the word “MY” in the context of the body. The profound consequences of the disease and the often radical treatment influence patients’ body image to a great extent [10]. As such, we wanted to determine if the patients felt integrated with their bodies during treatment or if they felt separate from their bodies and treated them objectively.

We differentiated the analysis based on the treatment stage to compare the quality and quantity of words uttered by patients and focused on the sentiment and number of adjectives in each stage. Adjectives were explored since they constitute a group of words that carry meaningful information about a subject or object and may express some emotional value. This was achieved by developing a rule to extract text containing adjectives related to the patient’s body. The next step focused on recognizing all negative and positive adjectives in the matched notes. The positive–negative recognition was based on the Nencki Affective Word List (NAWL) proposed by researchers from the Nencki Institute of Experimental Biology. The word list is a standardized database of 2902 Polish words selected based on the Berlin Affective Word List (BAWL), a standardized database of German words for studying emotions and other linguistic issues [11].

We assumed that matched positive and negative terms were qualitative adjectives used to describe nouns (in our context, the chief noun was BODY). In this class, attributive adjectives occur before a noun, and predicative ones come after the noun. The remaining adjectives were not analyzed, as they constituted possessive, distributive, and demonstrative classes.

The analysis used SAS Viya^®^ software v. 3.5(SAS Institute Inc., Irvine, CA, USA) [12] with an open architecture that allows data management, modification, and restoration from multiple sources. The first part of the analysis included uploading the text corpus to the platform, text parsing (excluding the terms non-essential for further analysis), stemming words (tokens) into their basic form, and calculating the desired word classes (verbs, past and present tense verbs, and positive and negative adjectives). The research also focused on detecting characteristic words indicating the most problematic areas for the patients. These terms referred to their face, skin, and hair and those signaling concerns over losing an attractive appearance.

#### 2.2.2. The Sentiment-Based Approach in Body Image Analysis

Patients diagnosed with head and neck cancer face a significant risk of experiencing body image disturbances due to the noticeable disfigurement that arises from primary cancer and its treatment [13]. The study aimed to investigate the sentiment of such disfigurement on patients’ body image using a hybrid model approach to weigh the sentiment derived from the dictionary of emotions and the deep recursive network machine learning algorithm. As previous authors noted [14], sentiment as a part of NLP traditionally classifies textual data as positive, neutral, or negative. However, our approach compared the experimental results with expert opinion to clarify whether the analysis included ambivalent sentiment (positive–negative). We assumed the traditional model of sentiment based on terms from the general sentiment vocabulary created by Wilson, Wiebe, and Hoffman, a database of nearly 8000 terms with specific polarity (positive, negative, or neutral) [15]. In the dictionary-based part, the authors adjusted the dictionary to the Polish language, as its original version was developed in English.

The machine learning part used a deep recursive network with five hidden layers using LSTM (Long Short Term Memory) cells. Applying LSTM works well with long sequence dependencies. As property vectors, we used 100-element pre-learned global property vectors developed according to the GloVe method, an unsupervised learning algorithm for learning vector representations of various terms [16]. Since our text corpus was relatively small compared to several other parameters used in artificial neural network models, we trained our model using the Stanford Amazon Dataset service [17], which contains 34,686,770 reviews of product and user information, ratings, and plaintext reviews.

#### 2.2.3. Identification of Potential Areas of Difficulty

For identifying potential areas of difficulty, such as topics identified as relevant to the psychological care of neck and head cancer patients by the specialist psychologist, the study used a computerized thematic modeling data analysis method, which reveals a specific theme or several themes in the analyzed documents. The study attempted to identify three areas of difficulty in notes made by patients, including body image, pain, and self-esteem. Dictionaries of positive and negative words specific to each topic were used for body image and self-esteem. For the topic of pain, only one dictionary was created.

Before analysis, each note was transformed according to the text mining (TM) method, lemmatization transformed words to their basic form, and punctuation marks were removed [18]. Next, each note was presented as a vector with a “bag-of-words” feature to determine how many times each word occurred in the document. A frequency matrix presented the dataset prepared in this way. The matrix contained rows of information about individual words occurring in all documents, and each column represented a particular document. The intersection of a row and a column contains information on the frequency of a given word in a given document. The dimensionality of such a matrix can be large, so TM uses a latent semantic analysis (LSA) based on singular value decomposition (SVD) to reduce the dimensionality of the resulting matrix [19,20]. The matrix obtained using this method has smaller dimensions, which can be interpreted semantically [21]. The matrix represents a specific multidimensional space; each dimension contains keywords representing one of the topics, and each word has a particular place in the space and a certain value. By analyzing the vocabulary in each note, it is possible to determine the value of each note’s belonging to each theme in a negative and positive context. With these affiliation values determined, the relative difference between the values was calculated and expressed as a percentage. Therefore, within a given topic, one of four results can ultimately be obtained (only for body image and self-esteem):

- A topic is included in a note in a negative context if the value of the topic affiliation in the negative context is greater than the value of the topic affiliation in the positive context and the relative difference is greater than 20%.
- A topic is included in a note in a positive context if the value of belonging to the topic in a positive context is greater than the value of belonging to the topic in a negative context and the relative difference is greater than 20%.
- The note refers to the topic in a mixed (positive–negative) context if the relative difference between the affiliations is less than 20%.
- The topic does not appear in the note if the value of belonging to this topic in each context equals 0.

For the topic of pain, the method only determined if the topic was raised or not.

The results obtained from the computational method were verified by a psychologist who rated each note by assigning it one of four flags (only for body image and self-esteem): 0—the topic was not mentioned in the note; −1—the topic was mentioned in a negative context; 1—the topic was mentioned in a positive context; 2—the topic was mentioned in a mixed (positive–negative) context.

For the topic of pain, the expert psychologist only used two flags: 0—the topic of pain was not mentioned in the note; and 1—the topic of pain was present in the note.

#### 2.2.4. Emotion Measurement Method

**Figure 1:**
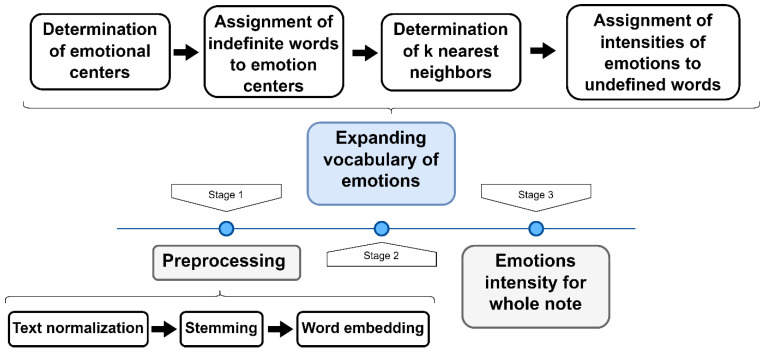
Diagram of the emotion measurement algorithm.

The next step in the analysis of the patient’s text notes was to detect five main emotions, including happiness, sadness, fear, anger, and disgust. The basis for defining emotions was the NAWL dictionary. In the collected corpus, only 10% of the words were also in the NAWL dictionary. To obtain a fuller picture of emotions, we expanded the dictionary. The diagram below (Figure 1) shows the steps of the complete emotion determination algorithm.

The preprocessing stage involved text normalization (removing stop words and general standardization like lowercase letters), a stemming operation (the process of reducing words to their stem to unify them), and a word embedding operation (transforming words into numerical vectors to represent the relationship between words in the corpus based on the context of usage). The second stage focused on directory expansion by (I) determining the emotional center from the average of each dimension of words embedding with a particular dominant emotion, (II) assigning words in the corpus that are not in the NAWL dictionary to the most similar emotion centers using cosine similarity, and (III) determining emotion based on k-nearest neighbors (from the NAWL dictionary) calculated from Euclidean distance. The formula for each emotion calculation is as follows:

$$emo_{intensity}=\sum_{i=1}^{k}s_{k}\times w_{k}$$

where *s_k_* is the intensity of a certain emotion of the k-word and *w_k_* is the weight of the k-word based on distance from the analyzed word. The values of emotion intensity ranged from 0 to 1, where 0 means there is no emotion in the note and 1 is the maximum value of emotion in the note.

The final emotion values were calculated as the quotient of the sum of an individual emotion of all words with the same dominant emotion in a note multiplied by the number of words with that dominant emotion and the length of the note. More details on the method are included in a previous publication [22].

## 3. Results

### 3.1. Statistical Analysis of Word Types and Sentiment Tagging

The analysis included three parts. The initial part involved tagging and calculating different parts of speech referring to the patient’s body, including adjectives, verbs, and possessive adjectives such as “MY”. Regarding adjectives, the study aimed to identify the total tag count and classify them based on their positive or negative connotations at a particular treatment stage. The analysis of verbs followed a similar approach, with a focus on identifying references to the past tense. However, we excluded verbs and the term “MY” from the analysis in particular stages.

**Figure 2:**
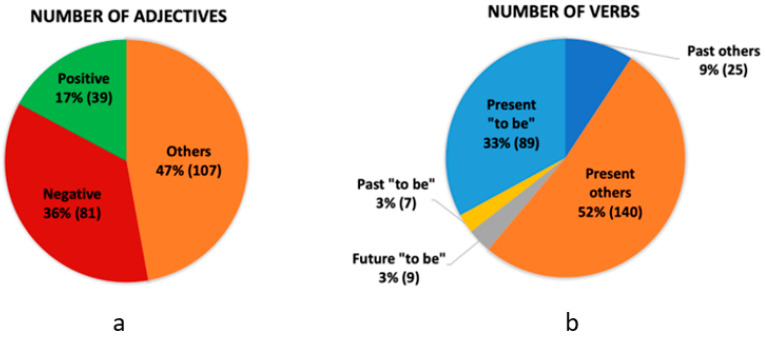
Statistical analysis of target words, including number of adjectives (**a**) and number of verbs (**b**).

The analysis presented graphically in Figure 2a revealed 227 adjective terms, of which 81 were matched as negative and 39 as positive. Further insight showed that both of these categories referred directly to body description. Patients very often used adjectives to describe their bodies, for instance, an ugly body, a painful body, or my body is strong. Therefore, we decided to extract positive and negative tokens out of all the adjective matches. The adjectives labeled as others had 47% matches among all terms. They represent terms giving general information on patients, such as eye color, hair color, reference to age, and similar conjunctions, demonstrative adjectives, and possessives. Regarding the verb analysis (Figure 2b), we identified 270 matches. The main focus was on detecting the verb “*to be*” in various tenses. Our data contained 105 matches of “*to be*”. Among these, 7 were in the past tense, 89 in the present, and 9 in the future tense. The verbs labeled “*present others*” and “*past others*” were 163, and they represent terms associated with states, actions, and perception. Within this category, 25 tags were in the past tense, and 140 were in the present tense.

Table 1 shows the mean percentage of matched adjectives per patient note length at a particular treatment stage, which demonstrates what part of the note constitutes negative and positive adjectives. In the first stage, the results showed a high saturation of negative adjectives (14.45%), while the second stage had a decrease in negative adjectives and the highest saturation of positive words. However, negative words increased in stage III compared to stage II.

The study aimed to compile a list of characteristic terms related to body image, and it was observed that patients frequently employed negative words, particularly when discussing their face, skin, and hair. These expressions indicated concerns over the loss of attractiveness. Patients often referred to their bodies as ugly, old, dry, skinny, wrinkled, sick, devastated, weak, and disgusting. The findings also showed that patients frequently expressed concerns about their skin, describing it as dry, transparent, pale, and rough, with rashes and allergic reactions.

The sentiment was tagged by an expert and by the SAS tool, assuming three points of polarity: positive (1), neutral (0), and negative (−1). When comparing the expert tagging to SAS results, we noticed 12 records tagged differently. The notes with different/error tagging concerned polarities 0, 1 and 0, −1. These discrepancies could have resulted from the fact that a note could contain positive and negative phrases concurrently. However, a more precise technique would need to be developed to confirm this hypothesis. The treatment-stage analysis was based on the sentiment values retrieved from SAS.

**Figure 3:**
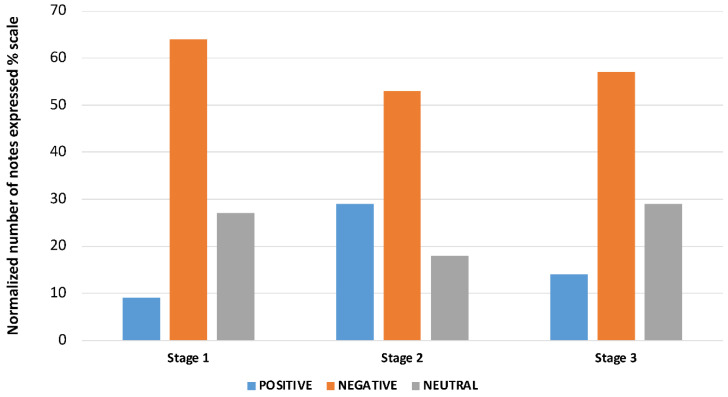
Sentiment analysis in particular treatment stages.

Figure 3 presents the number of notes in each stage, expressed on a percentage scale with sentiment tagging.

Based on 11 patient records, 9% of stage I patient notes had a positive sentiment, 27% had neutral polarity, and 64% were negative. The second stage encompassed 32 records, with 29% positive, 18% neutral, and 53% negative. The palliative stage (stage III) assessed seven records, of which 14% had a positive sentiment, 29% were neutral, and 57% were negative.

### 3.2. Analysis of Potential Areas of Difficulty

**Figure 4:**
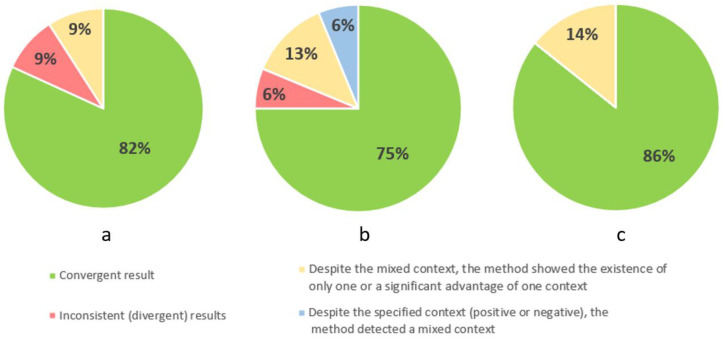
Percentage results of correct identification of the area of difficulty: body image in three stages of the illness: stage I (**a**), stage II (**b**), and palliative (**c**).

Figure 4 contains three pie charts showing the results for identifying the first area of difficulty, which is body image, at the three stages of illness. For this difficulty area, the method was consistent to the greatest extent for palliative patients (86%). Among these concordant results, a negative body image predominated (67%), a positive one occurred in 16.5% of cases, and a mixed context also occurred in 16.5%. The method found agreement for 82% of patients in stage I. Among the concordant results, negative body image prevailed (78% of cases), while positive body image accounted for only 22% of cases. For patients in stage II, the method was in agreement for 75% of cases, among which negative body image occurred in 46%, positive in 42%, mixed in 8%, and no topic was raised in 4% of cases. The method was incorrect in 9% of cases for stage I patients and 6% for stage II patients. In part of the notes, it was found that despite the mixed context indicated by the expert, the method showed an advantage for one of the contexts. This phenomenon occurred in each of the three stages of the disease. Despite a clearly defined context (positive or negative) for patients in stage II, the method showed a mixed context (6% of results).

**Figure 5:**
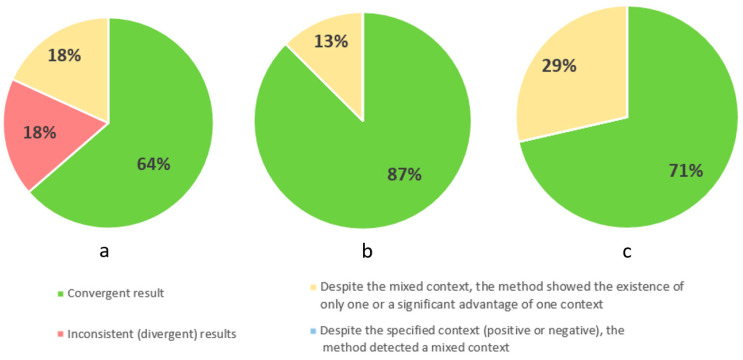
Percentage results of correct identification of the area of difficulty: self-esteem in three stages of the illness: stage I (**a**), stage II (**b**), and palliative (**c**).

Figure 5 contains three pie charts showing the results for identifying the second area of difficulty, which is self-esteem, at the three stages of illness. For this difficulty area, the method was consistent to the greatest extent for patients in stage II of the disease (87%). Among these concordant results, negative self-esteem was present in 46%, positive in 36%, mixed in 14%, and no topic was raised in 4% of cases. The method found agreement for 71% of palliative patients. Among the concordant results, negative self-esteem prevailed (80% of cases), while positive self-esteem accounted for only 20% of cases. The method showed the least concordance for patients in stage I of the disease, with a compliance rate of 64%. Among the concordant results, negative self-esteem prevailed (71% of cases), and positive self-esteem accounted for 29% of cases. For this topic, the highest percentages of cases were recorded when, despite the mixed context indicated by the psychologist, the computational method showed a predominance of one of the contexts. The highest percentage of such cases (29%) occurred for palliative patients. The method was incorrect in 18% of cases for stage I patients.

**Figure 6:**
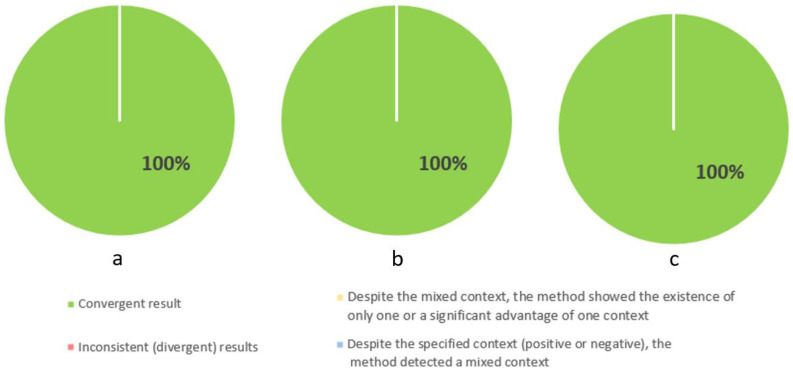
Percentage results of correct identification of the area of difficulty: pain in three stages of the illness: stage I (**a**), stage II (**b**), and palliative (**c**).

Figure 6 contains three pie charts showing the results for identifying the second area of difficulty, which is pain, at the three stages of illness. For this area, the method was in 100% agreement at each stage of the illness. This may indicate that this topic is the easiest to identify automatically using methods based on keyword dictionaries.

**Figure 7:**
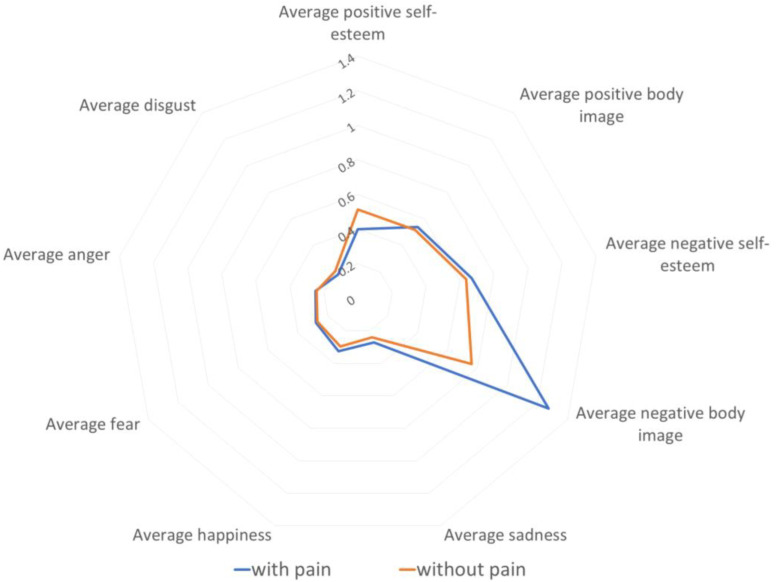
Radar chart of the mean values of two groups of patients with pain and without pain: negative body image, negative self-esteem, positive body image, positive self-esteem, sadness, happiness, fear, anger, and disgust.

Figure 7 shows a radar chart of the mean values of negative body image, negative self-esteem, positive body image, positive self-esteem, sadness, happiness, fear, anger, and disgust in two groups of patients, including those who mentioned pain in their notes and those who did not. The mean value of negative body image was predominant in those with pain and was significantly higher than in those who did not note pain. Patients indicating pain also had a slightly higher mean value of negative self-esteem. In patients with or without pain, the mean value of positive body image was approximately equal, though patients without pain had a higher mean positive self-esteem value.

People who experienced pain were characterized by greater sadness, happiness, and fear. Those without pain were characterized by a higher level of disgust.

### 3.3. Emotions Analysis

**Figure 8:**
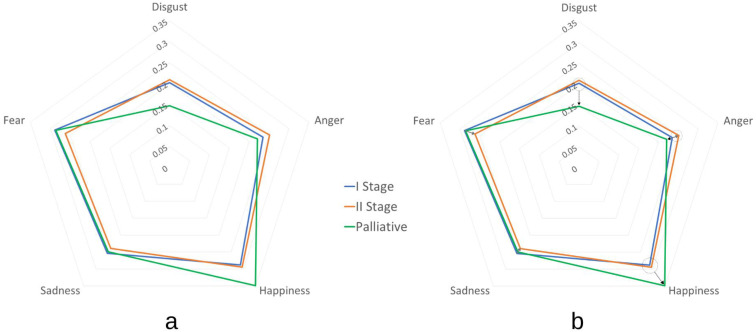
Emotional profiles of patients based on disease stage, including at diagnosis (*I Stage*), the treatment stage (*II Stage*), and palliative care (*Palliative*) (**a**). The emotional profiles of patients with marked transformations through disease (**b**).

Figure 8a shows emotional profiles for the patient groups at the diagnosis stage (stage I), treatment stage (stage II), and palliative care stage (Palliative). Anger level was higher in stage II patients than in stage I or during palliative care. The opposite occurred for fear, with the intensity of this emotion being higher in patients in stage I and with palliative treatment than patients in stage II. The prominent emotion is happiness, where patients with palliative care have the highest intensity. In feeling the emotion of sadness, a similar level occurs in patients in stage I and during palliative care, and slightly lower sadness occurs in patients in stage II. Stages I and II had similar levels of disgust, which were lowest in the palliative care group. The results are consistent with the professional opinion of psycho-oncologists and demonstrate gradations in the intensity of emotion depending on the stage of treatment.

Figure 8b shows the transformations of emotions depending on the disease stage. Solid gray arrows present an emotional change from stage I to stage II. As a result of the implemented therapy and the patient’s mental transition from stage I to stage II, a reduced level of sadness and fear is visible, with a simultaneous rise in anger levels. Dotted circles with black arrows indicate transformation from stage I or II to the stage of palliative care. There is an obvious reduction in the level of disgust and an increase in the level of happiness.

### 3.4. Statistical Analyses—Body Image, Self-Esteem, Emotions, and Stages of Diseases

Comparing body image assessed by an expert and the developed method demonstrated a high Kappa coefficient (0.65) and statistical significance (*p* < 0.000001), indicating non-random agreement between ratings. Similar statistics were shown when comparing self-esteem assessed by the expert and the computational method (Kappa = 0.69 and *p* < 0.000001). However, there was a lower level of agreement between the expert and the method for sentiment tagging (Kappa = 0.56 and *p* < 0.000001).

The second part of the analysis compared between-group differences. Only the level of happiness significantly affected body image, with no differences between the groups for ambivalent or negative body image opinions. The only emotion differentiating the disease stage was disgust, with significant differences visible between stage I and palliative and stage II and palliative.

## 4. Discussion

Patients diagnosed with head and neck or upper gastrointestinal cancer often experience a range of psychological challenges, including anxiety, stress, depression, and mood deterioration. Cancer poses a significant threat to the mental health of patients, eliciting a multitude of negative emotions. Fear of the disease, persistent worries, and an overwhelming sense of mortality are commonly reported among patients. Additionally, cancers in these areas often impact physical appearance, leading to body image issues and further exacerbating negative emotions throughout the course of the illness. As cancer patients often face challenges such as malnutrition and the need for enteral nutrition support, it is reasonable to anticipate increased occurrences of body image disturbances and mood deterioration within this population.

The study findings mostly agree with conclusions from previous studies and psychologists’ observations in oncology wards. In the areas of self-esteem [23] and body image [24], patients responded as expected and had poor outcomes. Conversely, happiness was detected more often in people receiving palliative care. This finding remains in line with psychological observations but has not been mentioned in previous studies.

Oncological treatment is associated with physical changes that can significantly affect body image. These include, among others, hair loss, postoperative scars, and amputations. However, changes in appearance are not the only reason for such a substantial change in cancer patients’ self-perception. Chronic pain associated with disease and treatment also has a significant impact. A study [25] on patients with a history of breast cancer and lymphoedema after surgical treatment showed that experiencing pain increases dissatisfaction with one’s body and intensifies depressive symptoms.

Lower happiness rates in the early stages of the disease (stages I and II) may be related to a lack of acceptance [26], with uncertainty over disease development as the basis for lowering patient well-being [27]. Those undergoing palliative care move to the acceptance phase and may try to improve their functioning, which was captured in the respondents’ statements. The higher happiness rate in patients receiving palliative care is a novel finding compared to previous studies.

Language and related emotion analyses can be used for screening and early psychological intervention in body image or mood deterioration. The language analysis could be an indicator of a patient’s narrative and may predict future behavior. The narrative construction of reality occurs by activating narrative schemes [28], which are responsible for interpreting situations and messages encountered and affect selectivity by ordering and arranging history [28]. Narrative thinking can adopt different styles and predict possible attitudes toward difficult situations. NLP and narrative analysis could be a tool for screening information on patients at the highest risk of mental disorders and may allow for a better selection of questionnaire screening methods.

Moreover, such language analysis could be used in mental health screening for other healthcare professionals and not only for psychologists. Indeed, training nurses, attendants, or other hospital staff in NLP data collection and transferring findings to psychologists for interpretation could be a source of information for doctors when determining the need for psychiatrist consultation and/or therapy and for triage.

The software used obtained divergent consistency of interpretation for different groups of patients. For those in the first stage of the disease (64%), the method did not allow conclusions to be drawn about patients without the full supervision of a specialist. For patients in stage II (71%), the study observations were sufficient to inform psycho-oncologists and psychiatrists on sensitive areas (anger, sadness, disgust) and the selection of aid measures. In palliative care patients (87%), the high agreement found is a good basis for continuing research in this direction (unexpectedly high level of happiness detected) and may be a reason to revise the current selection of screening methods.

Some patients in stages I and II of cancer may show broadly understood symptoms of anxiety, low self-esteem, and self-loathing. At this point, a specialized course for nurses on the principles of effective communication with patients may prove useful and could ensure the appropriate exchange of information to alleviate the symptoms experienced by patients. The course would also include psychoeducation oriented toward accustoming patients to their bodies and being in the hospital.

Such a course should also include information on what issues should only be discussed with the patient in the presence of a qualified psychologist.

In subsequent studies, it will be worthwhile to include patients in remission who are not supported by the care system, despite the need for ongoing psychotherapeutic care. Regular follow-up of these patients may allow for the early detection of possible post-treatment complications and potential recurrence, which would facilitate faster intervention and appropriate action. Monitoring those in remission could also be beneficial for identifying risk factors and reducing the chances of future relapse.

Another group that should be included in future studies is that of those who have previously suffered from cancer and are now considered cured. They may experience Damocles Syndrome and/or symptoms of fear of cancer recurrence (FCR). Both terms are often used interchangeably in the available literature, with the fear of becoming sick again still felt by those who have recovered. A study [29] of a group of young women who had suffered from breast cancer in the past showed that 70% of respondents reported FCR. In addition, a quarter of the respondents cited a significant impact on mood. It was also found that younger women suffering from cancer may be at higher risk of FCR symptoms.

## 5. Conclusions

What is new in the context of NLP in this article? First, we explore innovative approaches to creating dictionaries that address areas of difficulty specific to different patient groups. Second, we analyze the use of vocabulary specific to a given patient population, which allows for a more precise understanding of their needs and challenges. Thirdly, we compare the intensity of basic feelings in different phases of the disease, which allows for a better understanding of the dynamics of emotional changes and allows for a more appropriate therapeutic approach. The computational method developed provides a potential avenue for efficient and non-invasive screening of patients for negative body image and decreased well-being. Furthermore, it offers the capability to assess patients with speech disorders without imposing additional burdens on medical staff. This tool has the potential to aid clinical psychologists, nurses, and doctors in their patient care duties during hospital stays. An automated screening method for disturbed body images could potentially save care time and costs in dedicated oncology units. In addition, NLP methods may enable the detection of patients requiring psychological support at an early stage and prevent the aggravation of mental disorders. Since the perception of one’s body may be related to self-esteem [30] and quality of life [31], it seems reasonable to use such an approach in clinical practice.

The findings of the current study suggest potential enhancements in the care of oncology patients. By using NLP, nurses or other health care professionals may have improved capacity to identify patients in need of additional attention or psychological intervention, facilitating more effective communication. The tool presented in this study could also facilitate more comprehensive nursing interviews, providing valuable patient information to medical staff. NLP analysis could also be used by oncologists to highlight topics they should discuss when consulting with patients. Moreover, oncologists could better identify those who require psychological and/or psychiatric support.

The results of this study may be useful to psychologists and psychiatrists due to the specific challenges related to mental health for those undergoing oncological treatment. The tool is likely to help specialists in the faster recognition of patients with increased susceptibility to developing disorders, which shortens the waiting time for intervention and has a significant impact on the course and outcome of therapy. The findings may also aid in choosing the appropriate treatment regimen while accounting for the individual needs and possible concerns of the patient.

Despite the imperfections of the NLP method, it seems reasonable to improve it and attempt to implement it, especially in overburdened healthcare systems. Overlooking psychological care for oncology patients is a common phenomenon. As such, there is a need for simple and quick mental health screening tools for use in oncology units. The solution presented in this study may help implement psychological screening, diagnosis, or early intervention and can also support comprehensive and interdisciplinary approaches to patient care.

## Figures and Tables

**Table 1:** Mean percentage of adjectives per patient note length.

	Negative Adjectives	Positive Adjectives
Stage 1	14.45%	1.83%
Stage 2	6.81%	4.30%
Stage 3	11.65%	3.61%

## Data Availability

Data is contained within the article.
